# DeCoaD: determining correlations among diseases using protein interaction networks

**DOI:** 10.1186/s13104-015-1211-z

**Published:** 2015-06-06

**Authors:** Mehdi B Hamaneh, Yi-Kuo Yu

**Affiliations:** National Center for Biotechnology Information, National Library of Medicine, National Institutes of Health, 8600 Rockville Pike, Bethesda, MD 20894 USA

**Keywords:** Disease–disease similarity, Information flow, Disease networks, Protein–protein interactions

## Abstract

**Background:**

Disease–disease similarities can be investigated from multiple perspectives. Identifying similar diseases based on the underlying biomolecular interactions can be especially useful, because it may shed light on the common causes of the diseases and therefore may provide clues for possible treatments. Here we introduce DeCoaD, a web-based program that uses a novel method to assign pair-wise similarity scores, called correlations, to genetic diseases.

**Findings:**

DeCoaD uses a random walk to model the flow of information in a network within which nodes are either diseases or proteins and links signify either protein–protein interactions or disease–protein associations. For each protein node, the total number of visits by the random walker is called the weight of that node. Using a disease as both the starting and the terminating points of the random walks, a corresponding vector, whose elements are the weights associated with the proteins, can be constructed. The similarity between two diseases is defined as the cosine of the angle between their associated vectors. For a user-specified disease, DeCoaD outputs a list of similar diseases (with their corresponding correlations), and a graphical representation of the disease families that they belong to. Based on a probabilistic clustering algorithm, DeCoaD also outputs the clusters that the disease of interest is a member of, and the corresponding probabilities. The program also provides an interface to run enrichment analysis for the given disease or for any of the clusters that contains it.

**Conclusions:**

DeCoaD uses a novel algorithm to suggest non-trivial similarities between diseases with known gene associations, and also clusters the diseases based on their similarity scores. DeCoaD is available at http://www.ncbi.nlm.nih.gov/CBBresearch/Yu/mn/DeCoaD/.

## Findings

### Background

Identifying similar diseases can help in understanding their underlying causes and may even hint at possible treatments, the importance of which is evidenced by the development of numerous algorithms: those by Hamaneh and Yu [1], Cheng et al. [2], Li and Patra [3], Zitnik et al. [4], Goh et al. [5], and Mehren et al. [6], just to name a few. However, the accessibility to disease-similarity searches is limited as there are only few programs available for calculating disease–disease similarities. MimMiner, introduced by van Driel et al. [7], uses literature text mining to assign pairwise similarity scores to diseases. DOSim [8] works based on the Disease Ontology (DO) [9] and semantic similarity. DiseaseConnect [10] combines disease-gene associations from different databases to build a disease-gene network and, for each disease pair, calculates a hypergeometric *P* value indicating the significance of the number of the genes involved in both diseases. MalaCards [11] uses both text search and gene sharing to link diseases. In this note, we introduce a new program DeCoaD to compute disease–disease similarities (correlations) based on a recently developed method [1].

Our method uses the information flow [12, 13] in a disease–protein network to calculate the similarity or correlation between any two given diseases that have gene associations. In such a network proteins are linked if they are known to interact, and each disease is connected to the protein(s) encoded by its associated gene(s). Based on the expected number of visits under a random walk model, for a given disease, the method assigns a weight to each protein in the network. The correlation between two diseases is defined as the cosine of the angle between their corresponding weight vectors. Additionally, the method introduces a probabilistic clustering algorithm that finds overlapping clusters of diseases (also represented by weight vectors), based on their correlations (for details please see [1]).

Using the method described, DeCoaD finds and reports diseases similar to a user-specified disease, the clusters that the disease is a member of, and its membership probabilities. It also provides an interface to Saddlesum [14] to run enrichment analysis, i.e. to find biological terms from an annotated term database (such as Gene Ontology (GO) [15] or KEGG [16]) that best describe the weight vectors. Our protein–disease network was created by combining the output of ppiTrim [17] and gene-disease association data from the Comparative Toxicogenomics Database (CTD) [18], North Carolina State University, Raleigh, NC and Mount Desert Island Biological Laboratory, Salisbury Cove, Maine (http://ctdbase.org/). ppiTrim processes iRefindex [19], which incorporates entries from all major protein interaction databases. Our protein–disease network will be periodically updated to reflect changes in the protein–protein interaction and gene-association data.

As described in [1], the correlation calculated by DeCoaD is based on disease-related genes and the involved biological processes, hence not necessarily a measure of phenotypic similarity. In this aspect our approach is somewhat similar to that of DiseaseConnect [10] but different from others (MimMiner, DoSim, and MalaCards) relying, at least partly, on text-search. The key difference between DeCoaD and other programs (such as DiseaseConnect and MalaCards) utilizing disease–gene relations is that it goes beyond shared genes and employs the whole disease–protein network to compute pairwise correlations. We have already shown [1] that the results of MimMiner and those of DeCoaD are complementary, and that linking diseases only based on gene sharing (as suggested by Goh et al. [5]) results in a small subset of our disease network. The goal of DeCoaD is not just to reveal disease–diseases similarities that are already implied in the literature or databases but also to find new links between diseases that call for experimental verifications.

### Usage

#### Input

The main user-provided input for DeCoaD is the ID of the disease of interest. This could be one of the diseases included in the network, in which case the ID has to be either in OMIM (Online Mendelian Inheritance in Man) [20] or MeSH (Medical Subject Headings) [21] format, or a “new” (not present in the network) disease. The list of the included diseases can be accessed by clicking on the link in the provided help content. If the disease ID is not included in the network, a list of associated genes must be given in the provided text box. Even if the disease is already present in the network, the user may enter a list of associated genes. In this case, however, the existing gene associations of the disease are ignored, unless they are entered in the text box. This enables the user to conduct *in silico* investigations on the impact of adding or removing gene associations or adding a new disease. However, such changes in the network require all the weight vectors to be recalculated, which results in an increase in the running time of the program. In addition to the input disease, the user needs to limit the number of reported similar diseases and clusters. This is done either by providing the lowest rank acceptable or by specifying the minimum correlation (for diseases) and the minimum membership probability (for clusters). When the lowest rank is specified and when there is a tie in correlations/membership probabilities, the program outputs more diseases/clusters than specified.

#### Output

**Figure 1:**
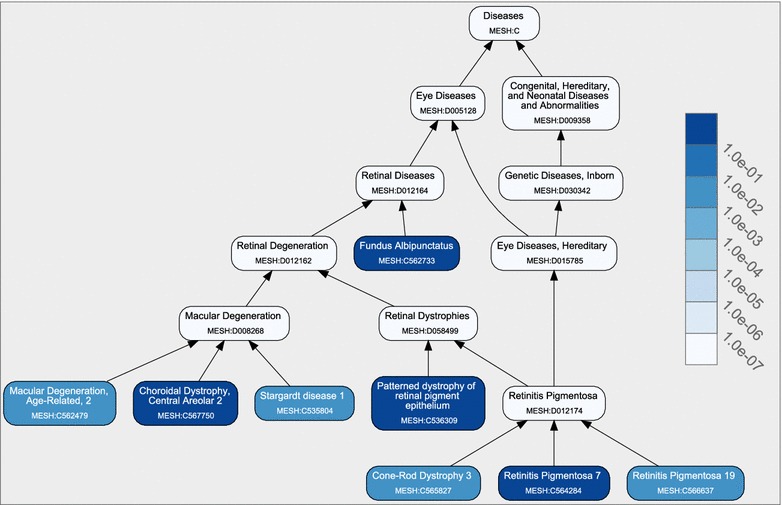
Graphical summary of DeCoaD. The graphical summary of the results when the input disease is Retinitis Pigmentosa 7 (RP7) (MeSH ID: C564284). For each disease represented by a leaf node, the *blue color* intensity indicates the correlation strength with the input disease RP7. The non-leaf nodes, always displayed in *white*, are never included in the calculation. They are only shown to reflect the curated hierarchical structure of the disease families containing the identified similar diseases (nodes in *blue*) in the CTD disease database.

The result page of DeCoaD has two main sections. The first section summarizes the results in three subsections:

1. *Graphic summary* A graphical representation of the results is given here. The CTD disease database [22] (http://ctdbase.org/) is used to show a directed graph whose leaves, colored in blue, are the disease of interest and the top ranking similar diseases. Figure 1 shows an example of such a graph. As shown in the legend of the figure, darker shades of blue correspond to higher correlations between the identified diseases and the disease of interest. DeCoaD only computes correlations of the input with other diseases at the most specific level (leaf nodes). The non-leaf nodes, always displayed in white, are not included in the calculation. They are only shown to reflect the curated hierarchical structure of the disease families containing the identified similar diseases (nodes in blue) in the CTD disease database. Each node (disease) in this graph is linked to its description in the CTD database.
2. *Similar diseases* In this part, the names of the top-ranking diseases and their correlations with the input disease are given. It should be noted that the reported correlations are generally very small due to high dimensionality, but our analysis has shown that scores larger than $10^{-6}$ can be considered significant [1].
3. *Clusters containing the disease* The list of cluster IDs containing the disease and the corresponding membership probabilities are given in a table here. Each cluster ID is linked to a web page that lists, in descending order, the membership probabilities of all diseases. It should be noted that, as mentioned before, when new gene associations are provided in the input page, the weights and probabilities have to be recalculated. To speed up this process, the probabilities are calculated approximately. In such cases another column, which gives an upper bound for the error caused by the approximation, is added to the output table.

The second section of the output page provides an interface to Saddlesum [14], an in-house enrichment analysis program. The user has the option to perform enrichment analysis for the disease itself or any cluster that contains it.

### Example

**Figure 2:**
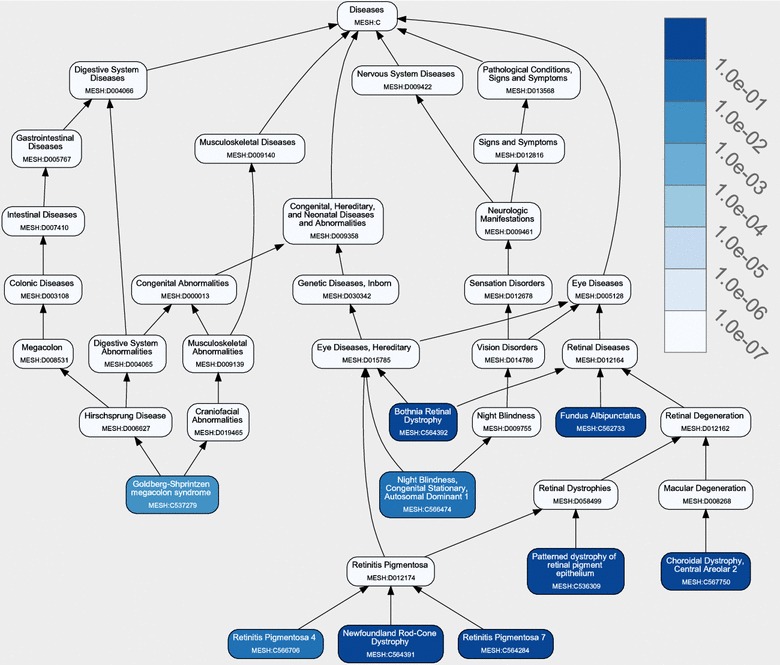
Another Graphical summary of DeCoaD. The graphical summary of the results when the input disease is Fundus Albipunctatus (MeSH ID: C562733). This input is one of the diseases reported as being similar to RP7 in Figure 1.

Figure 1 shows the first part (graphic summary) of the result page of DeCoaD when the input disease is Retinitis Pigmentosa 7 (RP7, MeSH ID: C564284). In this example, the correlation cutoff is set to 0.005. For comparison, Figure 2 shows the results with Fundus Albipunctatus (MeSH ID: C562733) (one of the diseases reported as being similar to PR7 in Figure 1) as the input disease (again the correlation cutoff is set to 0.005). The figure indicates that, although RP7 and Fundus Albipunctatus have a high correlation, DeCoaD results for these two queries are not identical. The difference between the results is due to the fact that the set of similar diseases given by DeCoaD depends on the user-provided cutoff, no matter what type of cutoff is used. Suppose that DeCoaD is run for the input disease $D_1$ with a correlation cutoff of $C_\mathrm{cutoff}$ and that diseases $D_2$ and $D_3$ are both found to be similar to $D_1$. This means that the correlation $C(D_1,D_2)$ between $D_1$ and $D_2$ is larger than $C_\mathrm{cutoff}$ and that $C(D_1,D_3)>C_\mathrm{cutoff}$, but these two facts do not guarantee that $C(D_2,D_3)>C_\mathrm{cutoff}$.

**Figure 3:**
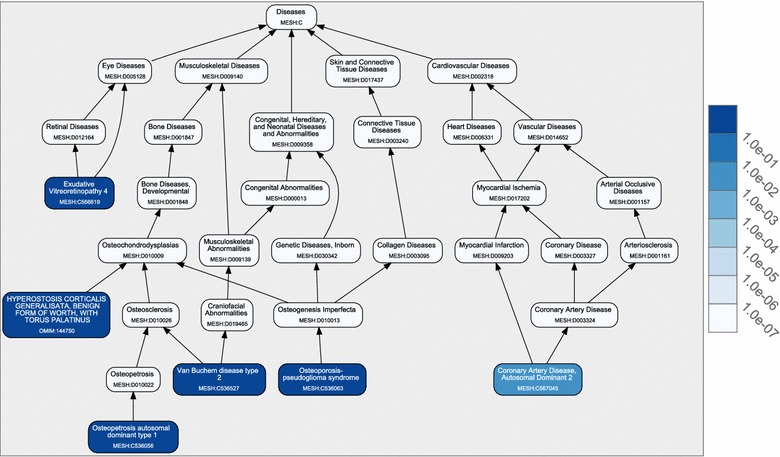
Diseases similar to Evr4. When Erv4 (an eye disease) is given as an input and the lowest rank cutoff is set to 5, the identified similar diseases are from a different family (musculoskeletal diseases). However, the diseases are all related to the Wnt signaling pathway.

**Figure 4:**
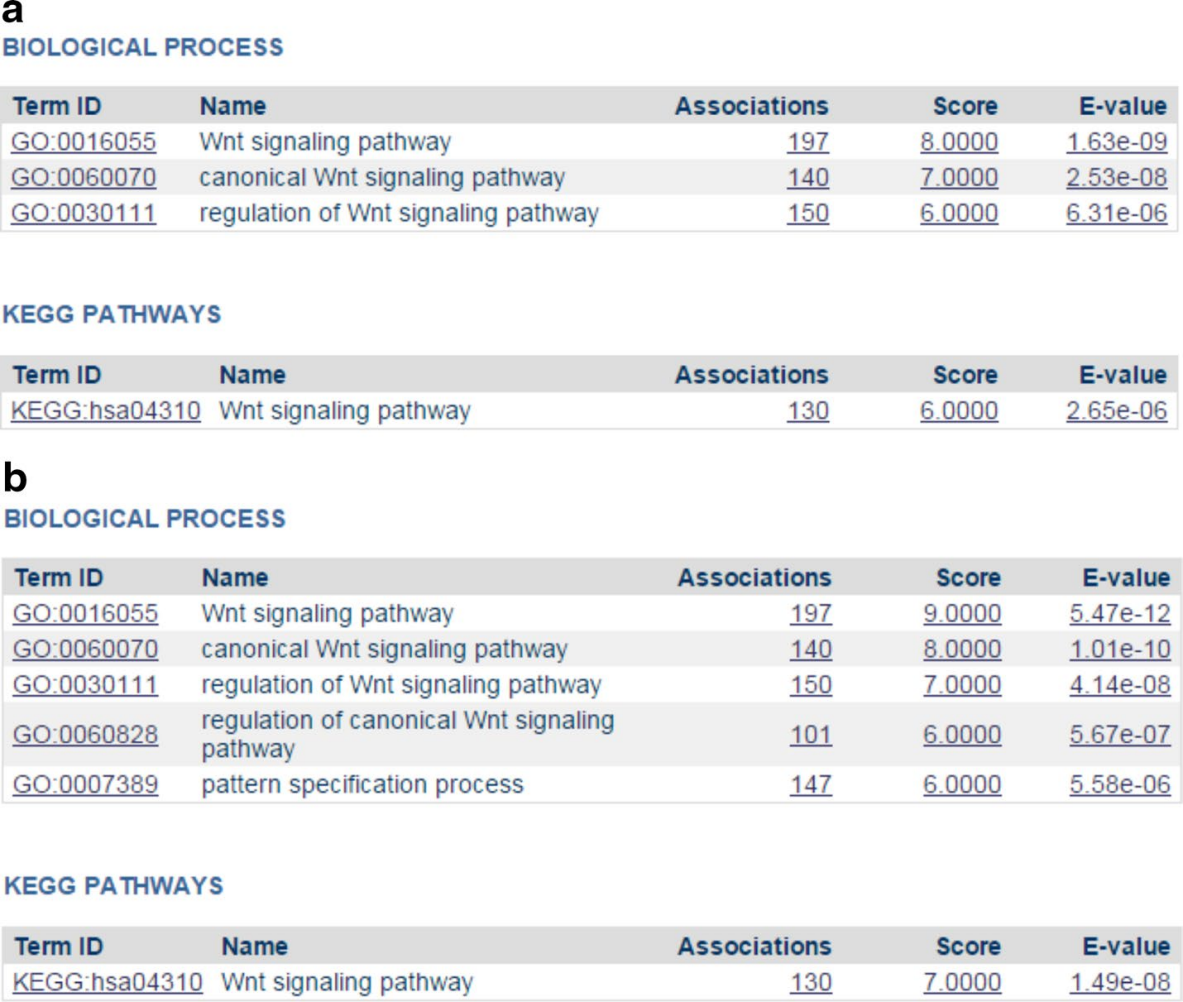
Enrichment results for Evr4 and the corresponding top ranking cluster. The top-ranking GO and KEGG terms found by the enrichment analysis are shown for Evr4 (**a**) and for the cluster that includes Evr4 with the highest probability (**b**). Although terms with *E* values less than $10^{-3}$ are deemed significant, we only display here terms with *E* values less than $10^{-5}$ to avoid crowdedness. The readers can see the whole list by running the SaddleSum interface on the DeCoaD results page.

**Figure 5:**
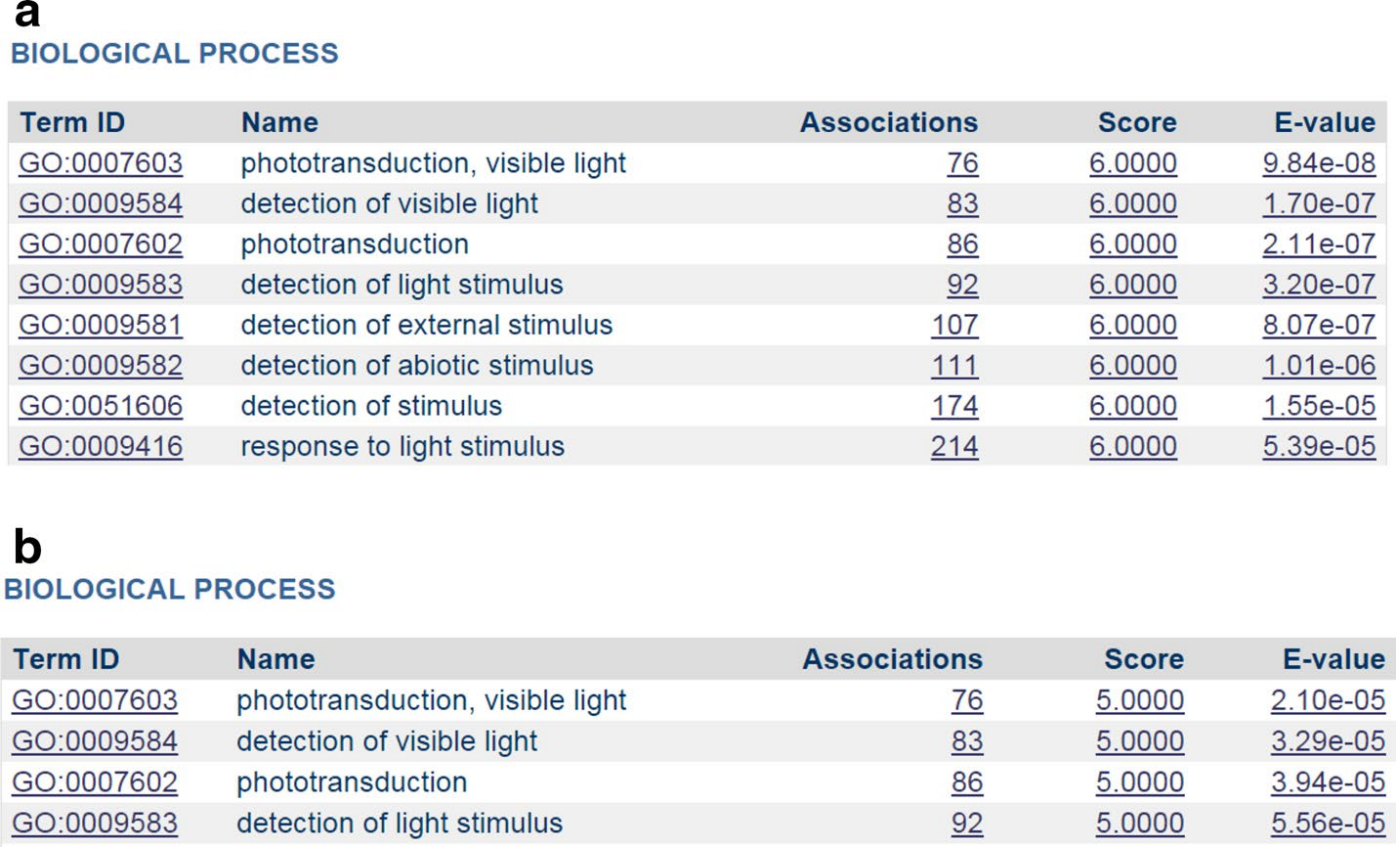
Enrichment results for the top ranking clusters associated with RP7 and Fundus Albipunctatus. The top-ranking GO and KEGG terms found by the enrichment analysis are shown for the top ranking clusters associated with RP7 (**a**) and Fundus Albipunctatus (**b**). Although terms with *E* values less than $10^{-3}$ are deemed significant, we only display here terms with *E* values less than $10^{-4}$ to avoid crowdedness. The readers can see the whole list by running the SaddleSum interface on the DeCoaD results page.

Figure 1 indicates that all identified diseases similar to RP7 are eye related. However, diseases found by DeCoaD are not always from the same family. As mentioned before, the disease–disease correlation calculated by DeCoaD is not necessarily an indicator of belonging to the same annotated family of diseases. Figure 3 shows an example of such a case when another eye disease, Exudative Vitreoretinopathy 4 (Evr4, MeSh ID: C566619), is used as an input. In this case, the identified similar diseases are not eye diseases, i.e. four out of five are musculoskeletal diseases and the fifth is a cardiovascular disease. Interestingly, all these diseases (and Evr4) have been reported to be related to Wnt signaling pathway [23], which is also the highest ranking term (with an *E* value less than $10^{-5}$) resulted from performing SaddleSum enrichment analysis for the weights associated with Evr4 and the two top ranking clusters that include it. In SaddleSum, the default cutoff *E* value is $10^{-2}$, but we choose to be more conservative here and regard terms with reported *E* values less than $10^{-3}$ as significant. Figure 4 provides some example results from such enrichment analyses. In comparison, Figure 5a, b show the results of the enrichment analyses when performed for the top ranking clusters associated with RP7 and Fundus Albipunctatus, respectively. The biological processes found by the enrichment analyses in these cases are related to phototransduction and light detection. It is worth noting that there is no guarantee that enrichment analyses will find significant terms for a given disease or cluster. However, as reported in our previous paper [1], Saddlesum is more likely to find term hits for clusters than for diseases. For example, using $10^{-3}$ as the *E* value cutoff, Saddlesum does not find any terms associated with either of RP7 or Fundus Albipunctatus, but it finds the terms shown in Figure 5 for the top ranking clusters associated with these diseases. This is an advantage of using our clustering method, which is discussed in detail in [1].

## Availability and requirements

- *Project name* DeCoaD.
- *Project home page*http://www.ncbi.nlm.nih.gov/CBBresearch/Yu/mn/DeCoaD/.
- *Operating system(s)* Platform independent.
- *Programming language* Python.
- *Other requirements* None.
- *License* All components written by the authors at the NCBI are released into Public Domain. Components included from elsewhere are available under their own open source licenses and attributed in the source code.
- *Any restrictions to use by non-academics* None.

## Abbreviations

CTD: Comparative Toxicogenomics Database
OMIM: Online Mendelian Inheritance in Man
MeSH: Medical Subject Headings
RP7: Retinitis Pigmentosa 7
Evr4: Exudative Vitreoretinopathy 4

## References

[1] Hamaneh MB, Yu Y-k (2014). Relating diseases by integrating gene associations and information flow through protein interaction network. PLoS One.

[2] Cheng L, Li J, Ju P, Peng J, Wang Y (2014). SemFunSim: a new method for measuring disease similarity by integrating semantic and gene functional association. PLoS One.

[3] Li Y, Patra JC (2010). Genome-wide inferring gene-phenotype relationship by walking on the heterogeneous network. Bioinformatics.

[4] Zitnik M, Janjic V, Larminie C, Zupan B, Przulj N (2013). Discovering disease–disease associations by fusing systems-level molecular data. Sci Rep.

[5] Goh KI, Cusick ME, Valle D, Childs B, Vidal M, Barabasi AL (2007). The human disease network. Proc Natl Acad Sci USA.

[6] Bauer-Mehren A, Bundschus M, Rautschka M, Mayer MA, Sanz F, Furlong LI (2011). Gene-disease network analysis reveals functional modules in mendelian, complex and environmental diseases. PLoS One.

[7] van Driel MA, Bruggeman J, Vriend G, Brunner HG, Leunissen JA (2006). A text-mining analysis of the human phenome. Eur J Hum Genet.

[8] Li J, Gong B, Chen X, Liu T, Wu C, Zhang F (2011). DOSim: an R package for similarity between diseases based on disease ontology. BMC Bioinform.

[9] Schriml LM, Arze C, Nadendla S, Chang YW, Mazaitis M, Felix V (2012). Disease ontology: a backbone for disease semantic integration. Nucleic Acids Res.

[10] Liu CC, Tseng YT, Li W, Wu CY, Mayzus I, Rzhetsky A (2014). DiseaseConnect: a comprehensive web server for mechanism-based disease–disease connections. Nucleic Acids Res.

[11] Rappaport N, Nativ N, Stelzer G, Twik M, Guan-Golan Y, Stein TI et al (2013) MalaCards: an integrated compendium for diseases and their annotation. Database (Oxford). Art. ID: bat018 10.1093/database/bat018 PMC3625956 23584832

[12] Stojmirovic A, Yu YK (2007). Information flow in interaction networks. J Comput Biol.

[13] Stojmirovic A, Yu YK (2012). Information flow in interaction networks II: channels, path lengths, and potentials. J Comput Biol.

[14] Stojmirovic A, Yu YK (2010). Robust and accurate data enrichment statistics via distribution function of sum of weights. Bioinformatics.

[15] Ashburner M, Ball CA, Blake JA, Botstein D, Butler H, Cherry JM (2000). Gene ontology: tool for the unification of biology. The gene ontology consortium. Nat Genet.

[16] Kanehisa M, Goto S (2000). KEGG: kyoto encyclopedia of genes and genomes. Nucleic Acids Res.

[17] Stojmirovic A, Yu YK (2011) ppiTrim: constructing non-redundant and up-to-date interactomes. Database (Oxford). Art. ID: bar036 10.1093/database/bar036 PMC3162744 21873645

[18] Davis AP, Murphy CG, Johnson R, Lay JM, Lennon-Hopkins K (2013). The Comparative Toxicogenomics Database: update 2013. Nucleic Acids Res.

[19] Razick S, Magklaras G, Donaldson IM (2008). iRefIndex: a consolidated protein interaction database with provenance. BMC Bioinform.

[20] Amberger J, Bocchini C, Hamosh A (2011). A new face and new challenges for Online Mendelian Inheritance in Man (OMIM). Hum Mutat.

[21] Coletti MH, Bleich HL (2001). Medical subject headings used to search the biomedical literature. J Am Med Inform Assoc.

[22] Davis AP, Wiegers TC, Rosenstein MC, Mattingly CJ (2012) MEDIC: a practical disease vocabulary used at the Comparative Toxicogenomics Database. Database (Oxford). Art. ID: bar065 10.1093/database/bar065 PMC3308155 22434833

[23] Johnson ML, Rajamannan N (2006). Diseases of Wnt signaling. Rev Endocr Metab Disord.

